# Anti-Drug Antibody Response to Therapeutic Antibodies and Potential Mitigation Strategies

**DOI:** 10.3390/biomedicines13020299

**Published:** 2025-01-26

**Authors:** Erin L. Howard, Melanie M. Goens, Leonardo Susta, Ami Patel, Sarah K. Wootton

**Affiliations:** 1 Department of Pathobiology, Ontario Veterinary College, University of Guelph, Guelph, ON N1G 2W1, Canada; ehowar09@uoguelph.ca (E.L.H.);; 2 The Wistar Institute, Philadelphia, PA 19104, USA

**Keywords:** monoclonal antibody, anti-drug antibody response, adeno-associated virus (AAV), vectored immunoprophylaxis

## Abstract

The development of anti-drug antibodies (ADAs) against therapeutic monoclonal antibodies (mAbs) poses significant challenges in the efficacy and safety of these treatments. ADAs can lead to adverse immune reactions, reduced drug efficacy, and increased clearance of therapeutic antibodies. This paper reviews the formation and mechanisms of ADAs, explores factors contributing to their development, and discusses potential strategies to mitigate ADA responses. Current and emerging strategies to reduce ADA formation include in silico and in vitro prediction tools, deimmunization techniques, antibody engineering, and various drug delivery methods. Additionally, novel approaches such as tolerogenic nanoparticles, oral tolerance, and in vivo delivery of therapeutic proteins via viral vectors and synthetic mRNA or DNA are explored. These strategies have the potential to enhance clinical outcomes of mAb therapies by minimizing immunogenicity and improving patient safety. Further research and innovation in this field are critical to overcoming the ongoing challenges of ADA responses in therapeutic antibody development.

## 1. Anti-Drug Antibodies (ADA): What Are They and How Do They Interfere with Therapeutic Efficacy?

### 1.1. History

The use of antibodies as therapeutics began in the 19th century when Emil von Behring discovered that administering serum from tetanus toxin immunized animals protected tetanus toxin naive animals [1,2,3]. This practice, termed “serum therapy”, was used as treatment for many diseases, including tetanus, diphtheria, botulism, and rabies, despite the side effects such as serum sickness, hypersensitivity reactions, and the risk of transmitting blood-borne pathogens that came along with it [3]. The development of the hybridoma technology in 1975 by Köhler and Milstein, where mouse spleen cells are fused with a murine myeloma cell line to create immortalized cells capable of secreting antibodies, allowed for antibodies to be produced in large quantities outside of a host [2,3,4]. The first monoclonal antibody (mAb) developed using hybridoma technology was approved for therapeutic use in 1986. This mAb, called orthoclone OKT3, is a CD3 targeting murine antibody used for the treatment of transplant rejection [5]. This was followed by the development of several other therapeutic mAbs, also using the hybridoma technology, and started the era of popularized antibody therapy [4]. Strategies to reduce the immunogenicity of mAbs led to therapeutic mAbs becoming increasingly more humanized as new technologies were developed. The first mouse–human chimeric antibody was approved by the FDA in 1997, the first humanized antibody was approved in 2001, and the first fully human antibody was approved in 2002 [2,6].

Today, mAbs are used in many therapeutic regimens and to treat a wide range of diseases. mAbs are used to treat conditions such as rheumatoid arthritis, Crohn’s disease, hemophilia A and B, cancers, psoriasis, infectious diseases, and many more [6]. In 2021 the 100th mAb therapeutic was approved by the FDA [7], and in recent years, mAb therapeutics have accounted for 50% of new FDA biologic approvals [8]. The therapeutic antibody market was valued at almost 238 billion USD in 2023 and is expected to reach almost 680 billion USD by 2033 [9]. It is clear that antibody therapeutics are a promising area of development. However, despite efforts to reduce the immunogenicity of therapeutic mAbs, a large proportion of mAbs on the market still result in the production of ADA responses.

**Figure 1:**
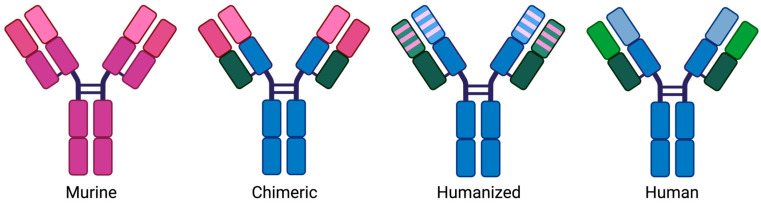
Murine, chimeric, humanized, and human antibodies are depicted in that order from left to right. Segments colored pink are of murine origin, and segments colored blue/green are of human origin. Created using BioRender.

Since antibodies produced from hybridomas are entirely mouse derived, they are foreign and thus can induce human anti-murine antibody (HAMA) responses in some patients [4]. To reduce the immunogenicity of these fully murine antibodies, the method of chimerization was introduced, whereby the constant domains were replaced with human immunoglobulin sequences, leaving only variable domains of mouse origin [2]. Eventually, the variable framework regions could be replaced by human sequences, leaving only the complementary determining regions (CDRs), which functioned as the antibody binding domains, as being of murine origin [4]. This greatly reduced the immunogenicity of mAbs; however, the remaining murine sequences still resulted in the development of anti-drug antibodies (ADA) against the chimeric antibody. The next step taken was replacing as much of the antibody gene as possible with human sequences in a process called humanization. Several strategies were used to achieve this end and were mostly focused on the removal of non-self-sequences, determined by comparing the murine antibody with its human homolog [10,11]. Since the CDRs are extremely important to antigen binding and therefore the function of the antibody, care must be taken to ensure that the sequences being replaced do not lead to decreased antigen binding affinity [4]. To this end, each residue that is identified as different between the murine and human versions is carefully evaluated for the effect it would have on antigen binding affinity if it were to be changed [12]. This process can be aided by in vitro phage display and yeast display techniques that allow for assessment of amino acid changes on binding affinity as well as by in silico platforms that allow 3D modeling and analysis of structure and homology of any prospective mutation [10]. The term CDR grafting is used when only the CDR regions of the antibodies are replaced, whereas editing of both the CDR and framework regions leaving only the specificity determining residues (SDRs) intact is called SDR grafting [4]. Another strategy, termed resurfacing, involves only the replacement of residues that are on the surface of the antibody after its secondary structure has been established [4]. Finally, fully human antibodies are now able to be developed using methods such as transgenic mice engineered to express only human immunoglobulin genes or fully synthetic or human donor B-cell-derived libraries [2]. For an overview of murine, chimeric, humanized, and human antibodies, see Figure 1. Two transgenic mouse models created in the 1990s, the HuMAb Mouse and the XenoMouse, were trailblazers in the production of fully human antibodies, but many other transgenic mice platforms have been developed since then [13]. These antibodies, which only carry sequences of human origin, should theoretically not produce any immune response in the patient. However, humanized and fully human antibodies have shown similar propensities to develop ADAs, and even some fully human antibodies are unable to overcome the issue of immunogenicity [2].

### 1.2. What Are Anti-Drug Antibodies?

Anti-drug antibodies are formed when the host immune system recognizes all or part of a biologic therapeutic as foreign and initiates an immune response toward the therapeutic. This is a unique aspect of biologic drugs that does not occur when small-molecule drugs are administered [14]. These ADAs can bind to the therapeutic mAb, potentially altering the pharmacokinetics and biodistribution by enhancing its clearance from the host, thereby decreasing the mAb concentrations in circulation or tissues [15]. This can lead to reduced efficacy of the therapeutic mAb or to hypersensitivity reactions and adverse events in the patient including anaphylaxis [15]. ADAs have been identified for a plethora of mAb therapeutics and have caused clinical trials to be discontinued and drugs to not be approved or to be withdrawn after approval [11].

### 1.3. Formation of ADAs

**Figure 2:**
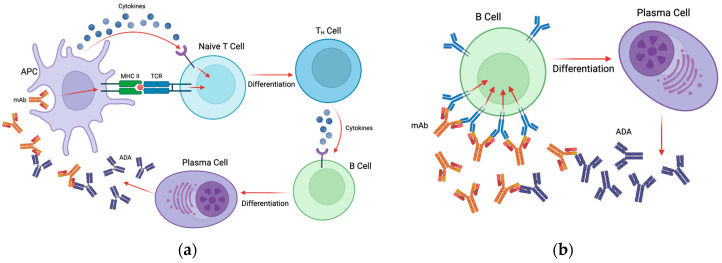
Overview of the process by which ADAs are formed. (**a**) In the T-cell-dependent pathway, APCs endocytose the therapeutic mAb and present linear epitopes in the context of MHCII to naive CD4+ T cells. APCs produce cytokines during antigen presentation that, along with the MHC–T-cell receptor (TCR) interaction, causing naive T cells to differentiate into CD4+ helper T cells. These activated T helper cells then release cytokines to stimulate B cells to differentiate into plasma cells and begin the production of antibodies against the therapeutic. (**b**) In the T-cell-independent pathway, therapeutic mAbs with multiple epitopes crosslink BCRs directly and stimulate differentiation into plasma cells that secrete drug-specific antibodies. Created using BioRender.

Anti-drug antibodies develop against therapeutic mAbs in either a T-cell-dependent or T-cell-independent manner. In the T-cell-dependent pathway, antigen presenting cells (APCs) such as dendritic cells (DCs) endocytose the therapeutic mAb and present linear epitopes in the context of MHCII to naive CD4+ T cells [16]. Additionally, APCs produce cytokines during antigen presentation that, along with the MHC-T cell receptor (TCR) interaction, cause naive T cells to differentiate into CD4+ helper T cells [16,17]. These activated T helper cells then release cytokines to stimulate B cells to differentiate into plasma cells and begin the production of antibodies against the therapeutic [16,17] (see Figure 2a). The peptides that are presented by MHCII to T cells are, appropriately, called T cell epitopes (TCEs) [16]. Antibodies produced in a T-cell-dependent manner are predominantly of the IgG isotype, which are characterized as long-lasting high-affinity immunoglobulins [17]. Some of the ADA producing B cells will differentiate into memory B cells, which persist long term and can rapidly respond to the same antigen upon a second exposure [16].

In the case of T-cell-independent activation of B cells, therapeutic mAbs with multiple epitopes are capable of crosslinking B-cell receptors (BCRs) directly and stimulating the secretion of drug-specific antibodies [17] (see Figure 2b). Because this type of activation does not induce the strong signals required for isotype switching, antibodies produced in this pathway are mostly of the IgM isotype, which have a shorter half-life and lower antigen affinity as they do not go through the affinity maturation process [17]. Similarly to TCEs, B cells recognize specific epitopes on antigens called B-cell epitopes (BCEs); however, these epitopes are restricted to the surface of the therapeutic protein and are usually conformational in nature [8,16]. The presence of both TCEs and BCEs on a therapeutic mAb can contribute to ADA development; however, mAbs have shown differing immunogenicity levels from patient to patient and between disease indications, suggesting that epitope presence is not the only factor that contributes to immunogenicity [18]. Many other factors related to the drug, the patient, and the dosing regimen can all contribute to the formation of an anti-drug response.

### 1.4. Factors That Influence Development of ADAs

Factors that contribute most significantly to the formation of ADAs can be divided into three categories: drug related, patient related, and regimen related [19]. As discussed earlier, drug-related factors such as mAb amino acid and structural properties can contribute to drug immunogenicity. Additionally, post-translational modifications (PTMs) can have a significant impact on mAb ADA development. Glycans on bioprocess-produced mAbs that do not match the human glycosylation patterns can be potentially immunogenic. This was shown with Cetuximab, a chimeric mouse–human IgG1 mAb that targets epidermal growth factor receptor (EGFR). This mAb, which is produced in a mouse cell line that expresses the gene for α-1,3-galactosyltransferase and contains mouse glycans, resulted in hypersensitivity reactions and pre-existing IgE antibody responses toward the murine α-1,3-galactose, whereas a version of Cetuximab (CHO-C225) grown in Chinese hamster ovary (CHO) cells, which lack α-1,3-galactosyltransferase and thus do not produce this glycosylation pattern, was shown to be less immunogenic [20]. Other PTMs, such as deamination, oxidation, and isomerization, may occur during the manufacturing process and can also influence the immunogenicity potential of mAb biologics [21].

Sometimes, the mechanism of action or antigen target of the mAb itself may contribute to its potential to produce an ADA response. An antibody therapeutic whose goal is to trigger an immune response toward its target antigen may in turn cause an immune response to be triggered toward itself. For example, bispecific anti-CD3 antibodies, which are used in cancer treatment to initiate an immune response to tumor cells, have been shown to induce cytokine release syndrome, a systemic inflammatory response that results in an immune response toward the therapeutic [22,23,24]. Additionally, mAbs that target cell surface proteins may cause an increased uptake of the mAb–antigen complex by APCs, resulting in an increased immune response to the therapeutic [24]. Alemtuzumab, which targets APCs based on their expression of CD52, is highly immunogenic, a trait that is thought to be attributed to the nature of its target antigen [18]. Conversely, mAbs that act by dampening the immune system, such as those targeting T-cell responses or APCs or depleting B cells, have a lower likelihood of immunogenicity [24]. Additionally, the intended use of a drug can influence its immunogenicity. For example, Rituximab, a cluster of differentiation 20 (CD20) targeting mAb, has been shown to have an ADA frequency of 1% in non-Hodgkin lymphoma, 4–11% in rheumatoid arthritis, and 26–65% in systemic lupus erythematosus [25]. These findings suggest that risk assessment of mAb immunogenicity might differ based on the disease being treated or the target of the mAb, and these aspects should be considered when designing strategies to decrease ADA formation.

It has also been demonstrated that mAb aggregation caused by production and shipping of mAb therapeutics can cause multiple B-cell epitopes to cluster, cross-link with BCRs, and lead to T-cell-independent ADA responses [18]. These aggregates may also trigger the T-cell-dependent pathway due to the innate immune response causing enhanced DC maturation and antigen presentation [26]. Aggregates have been shown to produce majority IgG1 isotype ADAs, with IgG2, IgG3, and IgM isotypes at a less frequent rate [19]. Additionally, the isoelectric point (pI) of the mAb therapeutic may contribute to its potential immunogenicity, with mAbs possessing a higher pI being more immunogenic, potentially due to stronger interaction with cell surfaces that are negatively charged, leading to increased uptake of the mAb and antigen presentation [27].

Patient genetic factors can impact the potential for an ADA response against a therapeutic mAb. Specific human leukocyte antigen (HLA) haplotypes carried by an individual may influence whether an ADA immune response will be triggered. The HLA, also known as the major histocompatibility complex (MHC), is responsible for encoding cell surface proteins that differentiate between self- and non-self-antigens. Type two HLA (HLA-II) is presented on the surface of APCs, and the HLA-II repertoire of each patient dictates whether an antigen will bind and be presented on the surface of APCs. HLA-II haplotypes are therefore likely involved in the process of ADA formation [28]. Several studies have investigated a correlation between the HLA-II haplotype and the occurrence of ADA for a particular therapeutic mAb. Five HLA-II haplotypes were found to have a correlation with the ADA response to Adalimumab, a TNF-α neutralizing mAb for the treatment of autoimmune disorders [27]. Three of these haplotypes were correlated with lower incidences of ADAs, and the other two were correlated with higher incidences of ADAs [27]. Correlation between the HLA-II haplotype and ADA formation was also seen for Rituximab, Atezolizumab, and Infliximab [28]. It is important to note that more research is needed in this area to elucidate if these correlations occur across a wider range of therapeutic mAbs and if there are different sets of risk and protective alleles for each mAb [28]. It also remains to be elucidated how HLA protective and risk haplotypes could be harnessed to mitigate the formation of ADA, but it represents a promising avenue of research. Another patient-related factor to consider is that patients may have differing endogenous levels of the drug target, which may lead to sizable amounts of unbound mAb in some patients. A correlation was seen between baseline levels of TNF-α and ADA response, where patients with lower baseline levels of the drug target were more likely to develop an ADA response to the mAb therapeutic and vice versa. It was speculated that unbound mAb was more likely to trigger an immune response than mAb bound to its target [29].

Regimen-related factors, such as the route of administration, administration schedule, and dose, have also been shown to affect the immunogenicity of mAbs. For example, using consistently high doses or increasing doses of mAb therapy can be used to induce a state of immune tolerance to the therapeutic [30,31]. These concepts are explored in Section 3.7. Additionally, the administration route and schedule may affect the formation of ADAs [5,14,32,33,34] as discussed in Section 3.10.

For an overview of the specific properties, targets, and corresponding anti-drug antibody data for therapeutic monoclonal antibodies used to illustrate key factors that affect the production of ADAs, see Table 1.

## 2. Mechanisms of ADA-Mediated Clearance of Recombinant mAbs

Once mAbs are recognized by the immune system and ADAs are formed, there are a variety of ways they can contribute to reduced efficacy and premature clearance of the therapeutic mAb. A significant factor in determining how the ADAs function is where they are binding on the mAb.

### Where on mAbs Do ADAs Form?

ADAs can develop against different regions on a therapeutic mAb, and this can have various outcomes depending on the binding site. ADAs mounted against the chimeric mAb Trastuzumab showed preferential binding to the variable regions of the mAb, which were of murine origin, including the CDR and framework regions [18,36]. Now, even humanized and fully human antibodies can result in the production of ADAs, as antibodies will always have some sequence variation in the CDR regions due to somatic hypermutation [37]. A correlation has been observed where antibodies with sequences closer to the germline will have a lower probability of eliciting an ADA response, while antibodies with sequences highly divergent from the germline have a higher incidence of ADA formation [38]. It was also found through the analysis of 93 different human-origin mAbs that the use of rare V alleles corresponded with higher incidence of ADA, suggesting that choosing common V alleles and antibody sequences with fewer mutations when selecting human mAb candidates would be helpful for reducing the immunogenicity of an mAb therapeutic [17]. This does not carry over to humanized mAbs, as they are already too divergent from the germline.

Antibodies that are specific to the antigen-binding portion of an mAb, or its paratope, are characterized by the lack of cross-reactivity with other antibodies. These can be neutralizing or non-neutralizing depending on their activities [37]. An abundance of studies have indicated that the majority of ADAs formed toward their therapeutic mAb were toward the variable region [32,37,38]. More than 94% of anti-Adalimumab antibodies were blocked by incubation with the antigen binding fragment of an Adalimumab antibody, indicating that the vast majority of the ADA response was anti-idiotypic [37]. Additionally, ADAs toward the human and humanized mAbs Adalimumab, Golimumab, and Certolizumab were more than 97% directed at the antigen binding region (Fab) [37]. More specifically, many studies have indicated that ADAs form against the CDRs, or paratope, which represent a large part of the mAb idiotype [37,39]. Unlike non-specific clearance through cellular endosomes, which is the mechanism associated with clearance of mAb drugs at the end of their life span, neutralizing ADAs lead to the clearance of therapeutic mAbs by antigen binding-mediated internalization and clearance [10]. Therapeutic mAbs recognized by neutralizing ADAs undergo either receptor-mediated endocytosis after binding of the Fab to the mAb or Fcγ receptor-mediated endocytosis if their Fc region retains the ability to bind to the Fcγ receptor [40].

Non-neutralizing ADAs are those that do not impact antigen binding, recognizing foreign murine sequences, polymorphisms (allotypes) in the constant domain, hinge regions, or different glycan patterns [37]. Unlike neutralizing antibodies, which are specific to the mAb therapeutic and therefore cannot be pre-existing prior to the initial drug treatment, non-neutralizing antibodies may be pre-existing in patients [37]. While they do not directly inhibit an mAb by binding to the paratope, non-neutralizing antibodies can lead to increased drug clearance, leading to a reduction in efficacy, and can interfere with effector functions on some mAbs whose functions are dictated in part by the Fc region [37].

In addition to neutralization, ADAs can facilitate increased clearance of the therapeutic by the formation of immune complexes between them and the therapeutic mAb. The formation of immune complexes is mediated by factors such as the structure of both the ADA and the therapeutic and the relative concentrations of both [41]. Depending on the size of the immune complex, it can either be phagocytosed by macrophages or initiate the formation of the complement cascade by recruiting C1q to the immune complex [41]. By their nature, mAbs are multimeric and are therefore able to form large immune complexes even if the ADAs are only against a single epitope, as that epitope is present twice within the antibody [41]. The activation of the complement by immune complexes causes a type III hypersensitivity reaction, and if the clearance mechanism becomes saturated, extra ICs can deposit on tissues and result in tissue damage and inflammation [42,43]. In fact, immune complexes have been suggested to be the culprit of many downstream adverse effects of mAb therapy [44].

## 3. Strategies to Prevent ADA Formation

**Figure 3:**
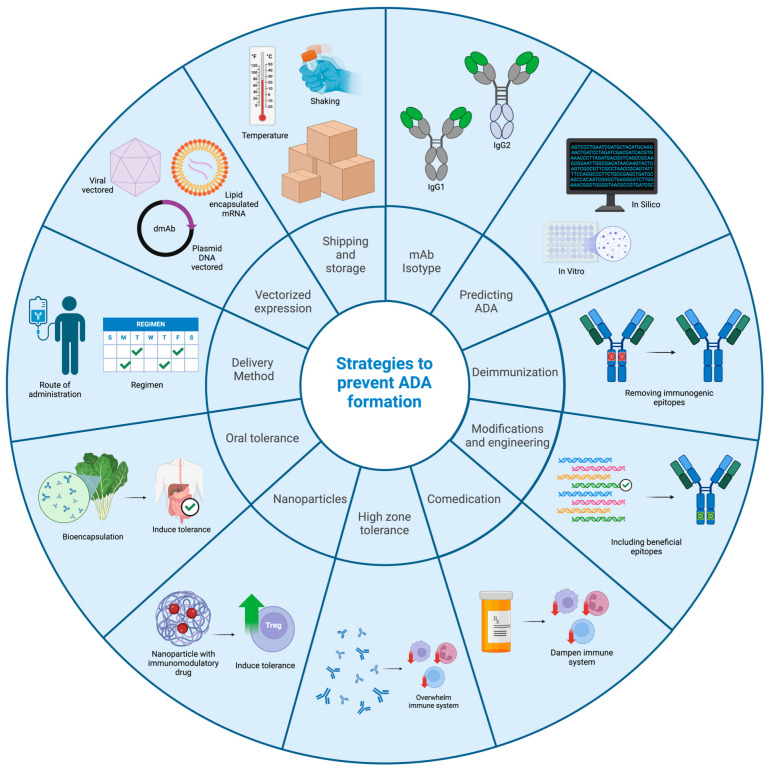
Overview of the strategies that can be used to prevent the formation of ADAs that are discussed in Section 3 of this work. Visual depictions of each category are provided in the corresponding segment of the wheel. Created in BioRender.

Anti-drug antibodies have been seen to some extent in almost every instance where their formation against therapeutic mAbs was investigated, and they were found to contribute to the development of side effects and diminished efficacy and have even led to the discontinuation of clinical trials. Therefore, strategies to mitigate the formation of ADAs are an extremely important area of research. In this section, many strategies for the mitigation of anti-drug antibodies will be discussed. For an overview of the strategies to prevent ADA formation discussed in this text, please see Figure 3.

### 3.1. Shipping and Storage

As discussed earlier, aggregation of mAbs formed during production, storage, and transit can stimulate the formation of ADAs [4]. Aggregates are formed when environmental stress conditions are applied to mAb drug formulations, such as temperature, shear forces, and movement [19]. A study investigating the effects of stress conditions on mAb drug formulations showed that when artificial stress conditions were applied to drug formulations, it led to the formation of aggregates [45]. The formation of small aggregations of mAbs acts as a molecular danger signal and can bind to pattern recognition receptors (PRRs) on immune cells such as monocytes and DCs, leading to their activation and maturation [19]. This produces a pro-inflammatory environment due to the production of cytokines and chemokines and can lead to further recruitment of immune cells [19]. It is therefore extremely important that protocols for the production, shipping, and storage practices are optimized to decrease the stress applied and therefore reduce aggregate formation, focusing on known stress conditions such as temperature, shaking, and shear stress. Recently, a study evaluated the formation of aggregates due to stress conditions when transporting five frequently used mAb therapeutics via pneumatic tube systems in hospitals, which involves changes in pressure, as well as mechanical movement of the tube. When mAb therapeutics are transported in hospitals, they are first diluted into infusion bags, which reduces the concentration of stabilizing compounds present and introduces air in the “headspace” of the infusion bag. Initially, the researchers noted that decreasing the “headspace” of the infusion bag was important as lower amounts of air/liquid interface resulted in decreased aggregation. Ultimately, this study revealed that the transportation protocols used in this hospital did not cause aggregation of the five tested mAb therapeutics [46]. The methods described in this study can be used to determine if other shipping and storage protocols cause aggregation in mAb therapeutics.

### 3.2. mAb Isotype

IgG1, IgG2, and IgG4 are the primary isotypes utilized for human mAb therapeutics due to their favorable serum half-life. Among these, IgG1 and its variants are the most common subclass with approximately 74% of approved IgG antibody therapeutics based on the IgG1 isotype, while IgG2 and IgG4 account for approximately ~13% each [47]. Despite the use of different IgG isotypes for therapeutic purposes, there is limited information in the literature about whether immunoglobulin isotypes carry differing risks of ADA formation. To the best of our knowledge, no head-to-head comparisons of recombinant mAbs that differ solely in their isotype have been conducted in humans. However, a study comparing rhesus IgG1 and IgG2 forms of four anti-HIV broadly neutralizing antibodies (bNAbs) delivered with an adeno-associated viral (AAV) vector to rhesus macaques showed that IgG2 mAbs elicited a lower quantity of ADAs than their IgG1 counterparts [48]. Additionally, when the animals were challenged with simian–human immunodeficiency virus (SHIV), none of the IgG2 immunized monkeys were infected compared with 67% of the IgG1 immunized monkeys [48]. These findings are important because IgG1 isotypes are currently the most widely used isotypes in mAb therapy but may not be the most optimal choice in terms of ADA responses, as indicated by the lower ADA incidence and greater SHIV protection provided by the IgG2 mAbs. More research needs to be done in this area to elucidate the immunogenicity of mAb isotypes and whether this aspect of mAb design can be used to decrease ADA formation toward mAb therapeutics.

### 3.3. Predicting ADA

With advancements in computing and laboratory technology, it is now feasible to use in silico prediction tools and in vitro assays at the beginning of the drug development pipeline to minimize time and money spent on drug products that are more likely to be immunogenic [49].

#### 3.3.1. In Silico Prediction Tools

T-cell epitope predicting algorithms were among the earliest in silico prediction tools used to investigate aspects of mAb immunogenicity [50] as TCEs consist of information that can be obtained directly from the sequence of the antibody [4]. However, when TCEs are predicted based solely on their sequence, some of the initially predicted sequences have been found to be non-immunogenic in vitro assays [8]. To overcome these limitations, improved TCE prediction tools were developed, leveraging the recent advancements in immunoinformatic processing power. These tools incorporate data from peptide–HLA binding affinities or HLA-II ligand data obtained through mass spectrometry [8,16]. Some examples of algorithms that use these tools include NetMHCIIpan and ISPRI [50], which have both been demonstrated to accurately predict immunogenicity of therapeutic mAbs [51]. TCPro is an immunogenicity risk assessment tool that compares a drug sequence with a given MHC-II allele distribution and can simulate the interaction between a biologic drug and immune cells [2]. Refinement of these prediction algorithms including advanced structural modeling will be important for furthering these approaches.

Unlike tools used to predict TCEs, BCE prediction tools need to be able to predict protein structures to identify conformational epitopes, which make up more than 90% of epitopes recognized by B cells [8,16]. BCE prediction tools must use higher levels of machine learning to predict how a protein will fold in 3D space, taking into consideration both conformational and linear epitopes and therefore what epitopes will be on the protein surface that could cause a B-cell-mediated immune response [8]. Factors such as hydrophilicity, flexibility, and tendency to form particular secondary structures need to be considered in order to accurately predict and identify BCEs [8]. BCE prediction tools use the physical properties of amino acids (such as BepiPred) or known regions of antibody binding (such as ElliPro and DiscoTope) to predict BCEs in therapeutic mAbs [16].

The Immune Epitope Database (IEDB) is one of the most widely used in silico prediction databases. It uses a large library of pre-established B- and T-cell epitopes and is a host for many algorithms and tools to predict highly immunogenic sequences within a protein [52]. The National Library of Medicine’s Basic local Alignment Search Tool (BLAST) is a frequently utilized in silico tool. Since the amount of sequence divergence from the human germline antibody sequence is positively corelated with the magnitude of an ADA response, BLAST can be used to predict potential immunogenicity through sequence alignment [3]. AbNatiV is a tool that uses deep learning to assess the likelihood that the variable fragment sequence of an antibody is similar to native human antibodies and therefore produces a score that predicts the immunogenicity of a given antibody [53]. This platform also offers a humanization platform that has been shown to produce humanized antibodies that retain or improve their binding capacity [53]. Tools such as SWISS-MODEL and ABodyBuilder2 can predict the 3D structures of mAbs, and programs such as AlphaFold and Phyre2 are able to predict the secondary structure even when a similar structure is not present in their database [8].

In silico tools powered by machine learning require training datasets with experimentally validated data, especially regarding conformational epitopes [8]. With additional experimental data and feedback into training algorithms, machine learning and artificial intelligence tools have the potential to further increase the accuracy of mAb predictions that can be utilized as a first step in the mAb therapeutic development pipeline.

#### 3.3.2. In Vitro Analysis of Immunogenicity

After in silico prediction of immunogenicity, it is important to verify the results using in vitro prediction assays. While in silico assays have become increasingly more accurate over recent years, they are not infallible, and therefore, many in vitro prediction tools are available to further narrow down therapeutic candidates.

Since anti-drug antibodies can be formed in a T-cell-dependent manner, monitoring the proliferation of T cells in vitro can provide accurate prediction of the immunogenicity of potential therapeutic mAbs. T-cell assays use peripheral blood mononuclear cells (PBMCs) derived from multiple healthy donors to predict immunogenicity by incubating with a library of overlapping peptides generated from the mAb sequence [54]. The proliferation of the PBMCs into CD4+ helper T cells is then monitored as an indication of immunogenic epitopes [16,54,55]. Similarly, CD4+ helper T-cell proliferation can be measured after exposure to donor PBMC derived DCs in a DC-T-cell coculture assay, which includes a restimulation step to make the test more sensitive [16]. DCs can also be used in the DC activation assay, which uses flow cytometry to detect the expression of cell surface markers of early DC activation following exposure to the mAb [56]. Alternatively, DCs can be used in MHC-associated peptide proteomics (MAPPs), where they are cultured with peptide libraries from the therapeutic protein, and the resulting MHC-II–peptide complexes are identified by mass spectrometry [55,57]. This assay is advantageous because of its ability to identify epitopes that are both processed and presented by immune cells [11]. MAPPs was successful at identifying epitopes that are known to be immunogenic in the mAb therapeutics Infliximab and Rituximab, and a positive correlation was seen between predicted immunogenicity and clinical ADA frequency in all six mAbs tested with MAPPs to predict immunogenicity [16].

The enzyme-linked immunosorbent spot (ELISpot) assay provides a readout of levels of cytokines specific to T-cell activation, such as IL-2 and interferon (IFN)-gamma, produced by the co-culture of DCs that were preincubated with an mAb to provide an idea of antigen-specific T-cell activation [58]. Flow cytometry can also be used to measure T-cell activation via the presentation of activation markers such as CD134 and CD137 on T-cell surfaces. This method correctly predicted the immunogenicity of bispecific and monospecific antibodies [51]. Flow cytometric identification of cells stained for proliferation markers carboxyfluorescein diacetate succinimidyl ester (CFSE) or synthetic nucleoside can be combined with activated T-cell markers to provide a clearer image of T-cell proliferation [59].

Epitope mapping strategies use DNA libraries of the target mAb with various single amino acid mutations to determine the epitopes that ADAs are binding to [16]. By introducing a variety of mutated versions of an epitope, it can be elucidated which residues are the most important in ADA formation. Mutated peptides that do not stimulate an ADA response can be sequenced to reveal the residues that are important in an ADA response, and steps can be taken to remove these residues and deimmunize the peptide [16].

A combination of the above assays can be used to accurately predict antigen immunogenicity and further validate the results from in silico prediction methods to select the most appropriate mAb therapeutic candidates [10].

### 3.4. Deimmunization

The use of in silico and in vitro prediction strategies discussed in the previous section can allow immunogenic epitopes to be discovered for mAb therapeutics. Deimmunization of protein therapeutics uses the removal of these immunogenic epitopes from the protein sequence and structure, resulting in a therapeutic that elicits lower ADA responses [16]. Many in silico prediction platforms also have tools to assist in the removal of problematic antigens without disrupting the function of the mAb therapeutic. An example of a computational strategy to help deimmunize proteins with minimal impact on function is called “dynamic programming for deimmunizing proteins”, which uses data on peptide–MHC-II affinity to predict T-cell epitopes and computational strategies to maintain functional properties of the therapeutic [35]. This process has been successful in deimmunizing several antibody therapeutics, with the predicted immunogenicity matching that of experimentally determined immunogenicity [35].

Some groups have been working on strategies to utilize the tolerogenic properties of regulatory T cells through adoptive transfer or ex vivo expansion of Tregs. Tregs from mice previously exposed to coagulation factor VIII (FVIII) were transferred to naive mice and resulted in reduced ADA titers upon challenge with an FVIII expression plasmid producing de novo protein, both at the initial transfer time and at 16 weeks post-transfer [60]. To produce antigen-specific Tregs against factor VIII, a T-cell receptor isolated from the T regulatory cells of a patient with hemophilia A was transduced into expanded human Tregs. These engineered Tregs were able to suppress the induction of T effector cells specific to FVIII in vitro [61]. Adoptively transferred Treg-induced suppression of ADAs has been shown to last more than two months after transfer, even when the transplanted Tregs are no longer able to be detected [62]. The use of exogenous Treg therapies has been investigated in protein replacement therapies, autoimmunity, and transplantation but may be applicable to mAb therapeutics as well [63].

Another strategy that utilizes T regulatory cells involves the incorporation of epitopes that stimulate T cells in a tolerogenic manor, termed “Tregitopes”, into the therapeutic design. Tregitopes are characterized by their ability to activate CD4+CD25+FoxP3+ T cells and were initially discovered when investigating TCEs. Tregitopes have been identified in several therapeutic proteins, as well as in the constant and antigen binding domains of immunoglobulins [64,65]. The discovery of Tregitopes has introduced the possibility of engineering therapeutic mAbs to actively induce tolerance to themselves. When these regulatory T-cell epitopes were incubated with PMBCs, this resulted in increased expression of regulatory chemokines, cytokines, and CD25/FoxP3 along with the activation of CD4+ T cells [18]. In vivo, Tregitopes have been shown to inhibit the proliferation of T cells by binding to MHCII in a way that mimics endogenous epitopes and reduces the production of effector cytokines, allowing for antigen-specific tolerance to be induced to the protein antigen [18,34]. A correlation between the presence of Tregitopes and absence of TCEs with a lack of immunogenicity was revealed in a retrospective study of therapeutic mAbs [64].

The solubility of the epitope has been shown to be important in the ability of the Tregitope to induce tolerance [34]. Therefore, Tregitopes have been designed as small peptides called apitopes, which have increased solubility and can be coadministered with therapeutics to induce antigen-specific tolerance [34]. Apitopes have been shown to bind exclusively to steady-state DCs and induce tolerance through the natural lack of costimulatory molecules present leading to a regulatory or non-inflammatory response. This causes T effector cells to convert to T regulatory Tr1 cells, resulting in the suppression of immune response gene priming [34]. By including these regulatory epitopes into the mAb coding sequence, the entire protein can be processed in a tolerogenic manner. Further investigation into the use of Tregitopes in mAb design needs to be conducted as this represents a promising avenue to produce self-tolerance-inducing mAb therapeutics [10].

### 3.5. Antibody Modifications and Engineering

The concept of rational design has been used to develop and deimmunize many antibody therapeutics. This concept combines knowledge of the function and structure of the therapeutic, as well as its interaction with other molecules, like those involved in the immune system, to inform modification of the mAb [8]. Another engineering technique involves directed evolution, a process that involves the creation of libraries of mutated mAb variants through an iterative process and high-throughput screening to select variants that display desirable properties. These include increased antigen binding and decreased immunogenicity [8].

As discussed in an earlier section, glycosylation patterns are important to the recognition of mAbs as self or non-self by the immune system. Engineering of mAb glycans to be less divergent from endogenously produced human Ig is another avenue with potential to reduce immunogenicity [32]. Several mutations have also been shown to help deimmunize mAbs by decreasing the likelihood that they will aggregate [2]. These include mutations such as the Ser35Gly mutation and mutations of regions identified by mass spectrometry to be sites of aggregation initiation [2].

### 3.6. Comedication

The coadministration of other medications is a commonly used strategy to reduce the likelihood that ADAs will form to a therapeutic mAb. A large portion of the drugs that are used in this application are of the immunosuppressive category. The concomitant administration of immunosuppressive drugs such as methotrexate and azathioprine has been shown many times over to reduce the production of ADAs in response to mAbs both when passively administered and when expressed in vivo from a viral vector [25,66]. Concomitant administration of methotrexate with Infliximab, as well as other TNF-α targeting mAbs, was shown to decrease the clearance of the therapeutic mAbs and reduce the extent of immunogenicity [67]. Increased concentrations of Infliximab and reduced ADA formation have also been shown when coadministered with azathioprine [32]. The administration of these drugs results in a transient immunosuppressed state for the patient and thus reduced the ADA response.

Other strategies have been investigated to induce a general suppression of the immune system at the time of mAb administration. Pre-treatment with mAbs that result in B-cell depletion is another way to inhibit the immune system [68]. The use of mAbs that target B cells, such as anti-CD20, anti-CD22, and anti-CD25, in conjunction with a therapeutic mAb has been shown to reduce the intensity of ADA responses to the therapeutic compared with treatment with the therapeutic alone [68]. Another avenue to be explored is the use of proteosome inhibitors, such as bortezomib, that have been used to reign in ADA responses that resulted from previous exposure to a therapeutic protein and resulted in successful immune tolerance upon further administration [11,30]. An advantage of using mAbs to achieve immunosuppression is that they possess greater specificity for their target cells, which decreases potential off-target effects when compared with small-molecule immunosuppressants [31].

However, the downside to these broadly immunosuppressive regimens is that by their nature, they result in a state of immunosuppression in the patient that is not always ideal. Additionally, they do not induce antigen-specific tolerance and would therefore need to be administered with every dose of the therapeutic mAb.

### 3.7. High Zone Tolerance/Immune Tolerance Induction/Drug Desensitization

A method that has been used for more than 40 years is “high zone tolerance” [11,69]. This method, also referred to as “immune tolerance induction” uses large doses of the therapeutic to overwhelm the immune system inducing a state of immune tolerance that is specific to the administered antigen [69]. Although the mechanism by which this strategy induces tolerance is not well understood, it is hypothesized that repeated high-dose exposure results in anergy and deletion of T cells specific to that antigen or that it induces T regulatory cells [11]. To support this idea, evidence has shown that an initially high level of mAb drug is predictive of a lower incidence of ADAs [32].

Similarly, drug desensitization is a process by which increasing doses of a therapeutic are given to a patient until a dose is reached that allows tolerance to the drug [70]. This line of treatment is used when a patient has already been exposed to the drug and produced an immune response toward it [70]. The mechanism of action for drug desensitization is also not confirmed, but it is theorized that the inhibition of mast cell degranulation and cytokine production play a role [30]. Strategies like these can be extremely helpful in allowing a patient to return to a previous treatment when no other options are available. However, since high zone tolerance and strategies like it require large doses of the therapeutic, they are extremely expensive. A single course of an immune tolerance induction protocol for rheumatoid arthritis patients receiving the blood clotting protein FVIII-inhibiting mAbs costs over USD 1 million [11].

### 3.8. Nanoparticles

Recent advances in the use of nanoparticle technology have opened a whole new avenue to reduce incidences of ADA responses to therapeutic proteins. Nanoparticles, self-assembling particles made of biodegradable polymers, are recognized by the immune system as virus-sized particles and are brought to lymphoid organs from peripheral tissues by resident APCs or are filtered from the blood by the liver and spleen [11,15]. Nanoparticles take advantage of this aspect of the immune system to effectively target APCs in lymphoid tissues. Often, nanoparticles encapsulate rapamycin, a compound that inhibits the mammalian target of rapamycin (mTOR) pathway and has been shown to induce regulatory T cells [15]. When rapamycin is delivered to immune cells it establishes a tolerogenic microenvironment that promotes processing of antigen in a tolerogenic manner [15]. Nanoparticles can be manufactured to co-encapsulate an immunomodulatory drug and the antigen to which tolerance is desired. This allows the antigen to be trafficked to immune cells along with the rapamycin, where it can be easily processed in a tolerogenic manner and result in robust antigen-specific tolerance [68]. However, rapamycin containing nanoparticles coadministered with the first dose of a therapeutic is also able to lead to tolerance toward the therapeutic [8,69]. When Adalimumab was administered with rapamycin nanoparticles, it successfully led to the prevention of ADAs in a mouse model [15]. Since the antigen is not encapsulated, it is not trafficked directly to the liver and spleen as the nanoparticles are and instead shows a broad biodistribution [15]. However, this method is still able to produce antigen-specific tolerance as some of the therapeutic is present in the immune tissues with the rapamycin nanoparticles and is processed in the same APCs, leading to the stimulation of Tregulatory cells that will then suppress any immune response toward the therapeutic mAb in peripheral tissues [15]. An advantage to coadministration is that no modifications need to be made to the therapeutic mAb, which would otherwise be required if it was encapsulated within the nanoparticle [15].

If the antigen and rapamycin do not need to be encapsulated together in the nanoparticle, why is it not possible for the rapamycin to be administered freely as well? Free (unencapsulated) rapamycin administered alongside therapeutics does not show an ability to induce tolerance toward the therapeutic [15]. Mice treated with ImmTOR nanoparticles, a brand name rapamycin encapsulating nanoparticle, alongside free therapeutic remained seronegative for ADAs against the therapeutic. Freely administered rapamycin and therapeutic showed no reduction in the ADA response when compared with control mice that were administered the therapeutic alone [15]. Interestingly, the total concentration of rapamycin administered via the ImmTOR particles was five times less than the amount administered freely [15]. However, in tissue culture, free rapamycin does not perform better than ImmTOR nanoparticles in terms of stimulating regulatory T cells [15]. The observation that rapamycin nanoparticles are better at inducing tolerance in vivo but not in vitro when compared with free rapamycin is likely due to the ability of the nanoparticles to preferentially traffic to lymphoid organs and provide a high local concentration in those areas [15].

A human proof of concept for the mitigation of ADAs has been demonstrated using SEL-212, a combination product composed of ImmTOR and pegadricase—a highly immunogenic enzyme therapy developed for the treatment of gout [71] but not for mAbs specifically. However, coadministration of the human anti-TNF-α mAb, Adalimumab, with rapamycin nanoparticles successfully led to the prevention of ADAs in a mouse model [15] and supports further evaluation of its effectiveness in human applications.

### 3.9. Oral Tolerance

Oral tolerance is another method that may be helpful in inducing antigen-specific tolerance to a therapeutic mAb. Oral tolerance is a naturally occurring phenomenon whereby the local immune system in the small intestine has evolved to process food antigens in a tolerogenic manner [72]. Harnessing this natural process to induce tolerance to therapeutic proteins is a promising avenue. To achieve this, the therapeutic would need to be delivered to the small intestine without becoming degraded by the acidic environment of the stomach [72]. One method that has been developed in this respect is to bioencapsulate the therapeutic by stimulating high production of the protein in the chloroplasts of plants that can then be consumed by the patient [72]. This was shown to be effective at reducing the incidence of ADAs toward protein replacement therapeutics for multiple diseases and could be a useful technique in mAb therapy [72]. It is important to note that when this method has been used to treat food allergies, it was observed that continuous therapy was needed for long-term desensitization to the target antigen. This may carry over to the use of oral tolerance in preventing ADAs, which might mean patients would have to continue injecting the target antigen for the duration of their mAb therapy [73].

### 3.10. Delivery Method

The administration method may also influence the immunogenicity of a therapeutic mAb. There has been some evidence to suggest that Infliximab, a TNFα-inhibiting mAb, is less immunogenic when delivered subcutaneously than when administered via infusions [33]. However, other studies have reported higher rates of immunogenicity from subcutaneous injections compared with intravenous routes [5]. For example, increased immunogenicity was seen for subcutaneous delivery of Tocilizumab and Trastuzumab when compared with intravenous delivery [5]. Given these conflicting results, more research needs to be conducted to determine if the mode of delivery of an mAb therapeutic affects its immunogenicity. If viable, this would be a simple aspect of mAb therapy that could be modulated to reduce the incidence of ADA.

Dosing patients on a regular schedule has been shown to lead to less immunogenicity than regimens that are episodic in nature [32,66]. In a systematic review and meta-analysis of ADA incidence to mAbs used to treat asthma, longer intervals between administrations and smaller drug doses were shown to increase the incidence of ADAs, but shortening the drug interval after ADA formation was not shown to be helpful in diminishing these already formed ADAs [14]. More research should focus on optimizing drug delivery regimens in an effort to identify a single regimen that can be applied to a broad range of therapeutics to reduce immunogenicity or to determine if regimens need to be tailored to a specific mAb therapeutic. This is of critical relevance for all mAbs as intravenous delivery routes are becoming more discouraged due to cost and logistical challenges including the need for controlled administration in a clinical setting, slow infusion (time) to reduce the likelihood of anaphylaxis, and additional post-infusion monitoring. Further strategies to reduce ADAs following subcutaneous or intramuscular administration will be of great benefit for mAb-based biologics.

### 3.11. Vectorized Expression of Therapeutic mAbs

**Figure 4:**
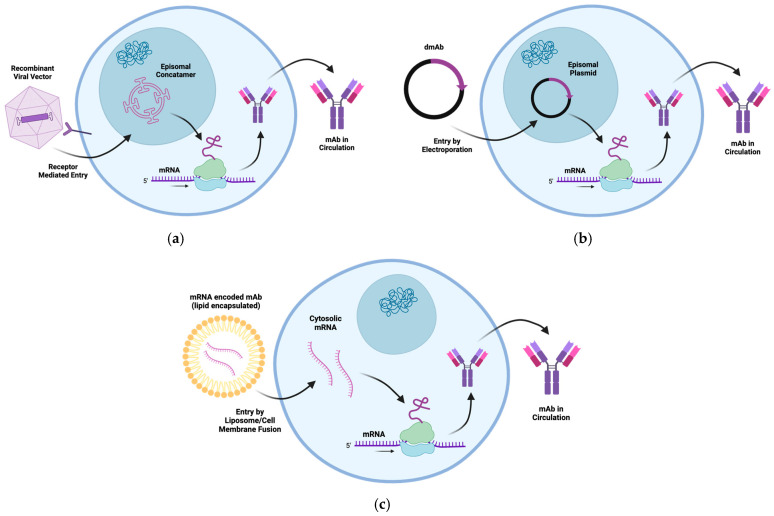
Overview of the process by which mAbs are produced in the host cell after delivery by (**a**) a recombinant viral vector, (**b**) a DNA-encoded mAb, and (**c**) an RNA-encoded mAb in lipid carrier. Created using BioRender.

Endogenous expression of therapeutic proteins involves producing these proteins within the patient’s body by utilizing the patient’s own cellular machinery. One way to endogenously express an mAb is by using a viral vector or nucleic acids (mRNA and DNA). For an overview of endogenous mAb expression methods, see Figure 4. Therapeutic immunoglobulin heavy and light chains can be encoded in AAV vectors, mRNA, or DNA for direct in vivo delivery. Through in vivo production, it is hypothesized that the mAb will incorporate post-translational processing endogenous to the patient instead of patterns derived from exogenous producer cell lines [74]. Currently, plasmid DNA mAbs can be targeted for local muscle expression. mRNA and AAV mAbs can be delivered to muscle, as well as other tissues depending on tropism. These cell and tissue tropisms have the potential to modulate mAb immunogenicity versus tolerogenicity. The liver, for example, has resident APCs, such as Kupffer cells and hepatic stellate cells, which process antigens in a nonconventional manner and lead to the expansion of T regulatory cells and anergy/apoptosis of effector T cells [3,74]. AAV vector capsids can be tailored to limit or expand host cell tropisms.

This can be achieved using liver-tropic viral vectors, such as AAV8 [74,75]. Indeed, AAV8-mediated delivery of mAbs to NHPs showed reduced immunogenicity when compared with AAV1-mediated delivery, the latter of which does not target the liver as efficiently [74]. A heterologous delivery strategy where NHPs first received an AAV8 vector expressing the therapeutic mAb followed by a second dose of AAV1 expressing the therapeutic mAb resulted in the highest mAb expression levels along with a diminished ADA response to the therapeutic [69,75]. Another possibility is to use viral vectors with B-cell targeting ability, such as AAV6, to be preferentially expressed there [74]. Most recently, an AAV8 vector encoding HIV bnAb VRC07LS was evaluated in a phase 1 dose escalation trial. Although some participants exhibited ADAs (three of eight participants) not all ADAs were neutralizing [76]. It is important to note that the use of a viral vector does introduce additional foreign components, the viral capsid and DNA, which may create a pro-inflammatory environment and increase the likelihood that ADAs will form to the transgenic mAb. Some studies have noticed that vectored delivery of an mAb, such as VRC01, results in higher ADA levels than when the same mAb is passively infused [48]. It is therefore important to consider the immune response toward the viral components when using vectors to deliver mAb genes and the use of alternative serotypes that may overcome this inherent immunogenicity.

Several preclinical studies have described mRNA-LNP antibody delivery and more recently safety and pharmokinetics in a phase 1 human trial. This has been shown using IVT-synthesized mRNA encoding the therapeutic tau antibody, RNJ1, whereby endogenous translation led to the development of functional full-length therapeutic antibodies when delivered to human neuroblastoma cells [77]. mRNA-encoded mAbs encapsulated in lipid nanoparticles provide more sustained expression. For example, mRNA antibodies against orthopoxviruses encapsulated in a lipid nanoparticle and delivered intravenously to mice were shown to be effective [78]. Among all current poxvirus therapeutics, antibody therapies for orthopoxviruses were shown to remarkably improve survival by reducing weight loss and tissue viral burden and successfully protected mice against severe disease. For a more in-depth analysis on mRNA-encoded mAbs and their applications in research, refer to reviews on the subject [79]. This mRNA–antibody approach was found to be simple and cost-effective and provided immediate protection, as well as flexibility [78]. For example, an mRNA–lipid nanoparticle encapsulating an anti-Chikungunya virus mAb (mRNA-1944) was evaluated in a phase 1 clinical trial, with no reported ADA development [80].

DNA-encoded mAbs utilize DNA as the template for antibody production. Once in the cell, the DNA is transcribed into mRNA and then translated into functional antibodies. In addition to the mAb half-life, as each DNA molecule gives rise to multiple mRNA transcripts, DNA-encoded mAbs can exhibit a long in vivo expression profile [81]. Additionally, due to the inherent stability of DNA as a molecule, it has also been shown to be more cold-chain independent due to its ability to withstand temperature fluctuations without incurring damage [81]. In addition to preclinical studies in small animals, recent data describing expression and protection in NHPs further support the approach of DNA-encoded antibodies [82], and a phase 1 clinical trial is underway [83].

The flexibility of mRNA-encoded and DNA-encoded mAb technologies also allows for rapid development and deployment, making them suitable for combatting emerging infectious diseases or rapidly mutating pathogens. Along with platform design and immunoglobulin sequence engineering, additional targeting strategies to further reduce ADA can be incorporated as part of the viral vector or nucleic acid design. MicroRNA binding sites can be incorporated into mAb expression constructs to further de-immunize predicted immunogenic sequences. This can be used endogenously to decrease the ADA response toward mAbs when they are expressed in vivo by a viral vector, mRNA, or DNA [75]. These microRNA bindings sites have complementary sequences to microRNAs that are highly expressed in APCs, which leads to reduced mAb expression in APCs and thus reduced MHC presentation of mAb peptides and, ultimately, a reduction in ADA formation toward the therapeutic mAb [75]. Together, these new gene-encoded approaches provide therapeutic delivery of mAb and strategies to overcome potential ADA through additional engineering.

## 4. Conclusions

The field of antibody therapeutics holds immense promise due to its ability to engage with a wide variety of targets with remarkable specificity. These therapeutics have the potential to become life-altering and life-saving treatments. However, their development has often been hindered by the formation of ADAs, which can lead to adverse side effects and reduce the drug’s efficacy by increasing mAb clearance. Therefore, it is crucial to understand the mechanisms behind ADA formation and to develop strategies to mitigate their occurrence in the context of antibody therapeutics.

Various strategies have been developed to deimmunize therapeutic antibodies, including the creation of humanized and fully human antibodies. Additionally, ongoing efforts are focused on developing prediction strategies, antibody engineering techniques, and immune modulation approaches. Researchers can employ a combination of these prediction, deimmunization, and immune modulatory techniques, as discussed in this review, to produce the most effective drug candidates with minimal ADA response.

Despite these advancements, further research is needed to explore new strategies for reducing immunogenicity as many antibody drugs on the market today continue to face challenges with ADA formation. Fortunately, recent research has begun exploring promising new avenues, such as the use of oral tolerance and tolerogenic nanoparticles, offering hope for the future of non-immunogenic antibody treatment regimens.

## Figures and Tables

**Table 1:** ADA responses associated with mAb-based products in humans.

mAb Name	mAb Format	mAb Target	Mechanisms Underlying ADA Responses	Reference
CD3xTRP1	Murine IgG2a, bispecific	CD3	Antibody therapeutics whose goal is to trigger an immune response toward its target antigen, may in turn cause an immune response to be triggered toward itself.	[22]
4D5-8, 225, 17-1A	Murine IgG1, chimeric IgG1, murine IgG2a, respectively	Her2/neu, EGFR, EpCAM, respectively	Dynamic programming for deimmunizing proteins was successful in deimmunizing these antibody therapeutics.	[35]
Cetuximab	Chimeric IgG1	Epidermal growth factor receptor (EGFR)	Glycosylation patterns on exogenously produced mAbs differing from host glycosylation patterns contribute to ADA formation.	[20]
Infliximab	Chimeric IgG1	TNF-α	Concomitant administration of methotrexate or azathioprine decreased clearance of the therapeutic mAbs and reduced extent of immunogenicity.	[32]
Infliximab	Chimeric IgG1	TNF-α	Higher serum levels of mAb drugs early in the therapeutic regimen are predictive of ADA-negative status for patients.	[32]
Infliximab	Chimeric IgG1	TNF-α	mAbs are less immunogenic when delivered subcutaneously than when administered via infusions.	[33]
Infliximab, Rituximab, and Atezolizumab	Chimeric IgG1, chimeric IgG1, humanized IgG1, respectively	CD20, PD-L1, TNF-α, respectively	Correlation seen between HLA-II haplotype and ADA formation.	[28]
Infliximab and Rituximab	Chimeric IgG1	TNFα and CD20, respectively	MAPPs was successful at identifying epitopes that are known to be immunogenic.	[16]
Rituximab	Chimeric IgG1	CD20	Varying frequency of ADA between disease indications for same drug.	[25]
Alemtuzumab	Humanized IgG1	CD-52	mAbs that target cell surface proteins may cause an increased uptake of the mAb–antigen complex by APCs, resulting in an increased immune response to the therapeutic.	[18]
Trastuzumab	Humanized IgG1	Human epidermal growth factor receptor 2 (HER2)	Preferential binding to the variable regions of the mAb, including the CDR and framework regions.	[36]
Trastuzumab and Tocilizumab	Humanized IgG1	HER2 and IL-6 receptor, respectively	Increased immunogenicity seen for subcutaneous delivery when compared with intravenous delivery.	[5]
Certolizumab Golimumab and Adalimumab	Humanized IgG1, human IgG1, and human IgG1, repsectively	TNFα	More than 97% of ADAs were directed at the antigen binding region.	[37]
Adalimumab	Human IgG1	TNF-α	Five HLA-II haplotypes were found to have a correlation with the ADA response.	[27]
Adalimumab	Human IgG1	TNF-α	Correlation seen between baseline levels of TNFalpha and ADA response. Patients with lower baseline levels of drug target more likely to develop an ADA response.	[29]
Adalimumab	Human IgG1	TNFα	Vast majority of ADAs were anti-idiotypic.	[37]
93 different mAbs	Human	Varying targets	Use of rare V alleles corresponded with higher incidence of ADA.	[17]

## Data Availability

No new data were created.
